# Diagnostic accuracy of the neutrophil-to-lymphocyte ratio and the platelet-to-lymphocyte ratio in rheumatoid arthritis: a systematic review and meta-analysis

**DOI:** 10.1007/s10238-024-01478-x

**Published:** 2024-09-04

**Authors:** Arduino A. Mangoni, Angelo Zinellu

**Affiliations:** 1 Discipline of Clinical Pharmacology, College of Medicine and Public Health, Flinders University, Adelaide, Australia; 2 Department of Clinical Pharmacology, Flinders Medical Centre, Southern Adelaide Local Health Network, Adelaide, Australia; 3 Department of Biomedical Sciences, University of Sassari, Sassari, Italy; 4 Department of Clinical Pharmacology, College of Medicine and Public Health, Flinders University and Flinders Medical Centre, Bedford Park, SA 5042 Australia

**Keywords:** Rheumatoid arthritis, Active disease, Neutrophil, To, Lymphocyte ratio, Platelet, To, Lymphocyte ratio, Diagnostic accuracy

## Abstract

Existing challenges with the early diagnosis of rheumatoid arthritis (RA) and active disease, mainly by non-rheumatologists, have prompted the search for novel biomarkers. Elevations in indices derived from blood cell counts, e.g., the neutrophil-to-lymphocyte ratio (NLR) and the platelet-to-lymphocyte ratio (PLR), have been reported in RA patients. However, their diagnostic accuracy has not been comprehensively assessed. Therefore, we conducted a systematic review and meta-analysis of studies reporting the sensitivity and specificity of the NLR and PLR, obtained by receiver operating characteristic (ROC) curve analysis, for the presence of RA and active disease. We searched electronic databases from inception to 15 March 2024 and assessed the risk of bias using the JBI Critical Appraisal Checklist (PROSPERO registration number: CRD42024533546). In 15 studies, the NLR exhibited acceptable accuracy for the presence of RA (area under the curve, AUC = 0.76, 95% CI 0.72 to 0.80) and active disease (AUC = 0.70, 95% CI 0.66 to 0.74). The PLR exhibited good accuracy for the presence of RA (AUC = 0.80, 95% CI 0.76 to 0.83). There were insufficient studies to assess the accuracy of the PLR for the presence of active disease. Our systematic review and meta-analysis suggests that the NLR and the PLR are promising biomarkers of RA (NLR and PLR) and active disease (NLR). Further research is required to investigate whether the NLR and PLR can significantly enhance the capacity to diagnose RA and active disease in clinical practice.

**Supplementary Information:**

The online version contains supplementary material available at 10.1007/s10238-024-01478-x.

## Introduction

A substantial body of evidence suggests that the early diagnosis of RA and the prompt recognition of increases in disease activity, i.e., active disease, in patients with confirmed RA are essential to prevent or minimize joint damage and disability and improve long-term outcomes [1–5]. However, the limited clinical experience in assessing these patients by non-rheumatologists, particularly in primary care, and the frequent overlap of mild signs and symptoms with other forms of inflammatory polyarthritis represent a significant barrier to accurate diagnosis [1, 5].

In addition to clinical and radiological assessment, several serological tests are available to assist physicians in diagnosing RA and increased disease activity. However, the diagnostic accuracy of such tests could be improved. For example, a systematic review and meta-analysis reported that the pooled sensitivity and specificity of the rheumatoid factor (RF) for the presence of RA were 69% (95% CI, 65% to 73%) and 85% (95% CI, 82% to 88%), respectively. Those of the autoantibodies against cyclic citrullinated peptide (CCP) were 67% (95% CI, 62% to 72%) and 95% (95% CI, 94% to 97%), respectively [6]. The use of conventional biomarkers of inflammation carries similar issues. For example, in a prospective cohort study conducted in primary care in the UK, the area under the curve (AUC) values of the C-reactive protein and the erythrocyte sedimentation rate for the diagnosis of RA were considered less than acceptable, 0.69 (95% CI 0.67 to 0.71) and 0.69 (95% CI 0.67 to 0.71) [7, 8].

In the search for novel, more accurate biomarkers of RA and active disease, several haematological indices derived from blood cell types assessed in routine blood cell counts, particularly neutrophils, platelets, and lymphocytes, are gaining increasing interest as markers of inflammation. Their easy determination and negligible costs have allowed their investigation in different disease states. Two such indices, the neutrophil-to-lymphocyte ratio (NLR) and the platelet-to-lymphocyte ratio (PLR), are significantly elevated in several immunological diseases [9–12]. Although similar elevations have been reported in association with RA and RA patients with active disease [13–15], a critical assessment of the diagnostic accuracy of the NLR and PLR in this patient group has not been conducted.

Therefore, we conducted a systematic review and meta-analysis of studies reporting the sensitivity and specificity values of the NLR and PLR from receiver operating characteristic (ROC) curve analysis for the presence of RA and active disease.

## Methods

### Literature search

We conducted a systematic literature search for articles published in PubMed, Scopus, and Web of Science from inception to the 31st of March 2024, using the following terms: “rheumatoid arthritis” and “neutrophil to lymphocyte ratio” or “neutrophil-to-lymphocyte ratio” or “NLR” or “platelet to lymphocyte ratio” or “platelet-to-lymphocyte ratio” or “PLR”. Two investigators independently screened individual abstracts and full articles according to the following inclusion criteria: (a) studies reporting the diagnostic accuracy of the NLR and PLR by sensitivity and specificity, obtained by receiver operating characteristic (ROC) analysis, for the presence of RA and/or active disease, assessed using conventional tools such as the Disease Activity Score-28 (DAS28) [16], (b) adult participants, and (c) full-text availability of articles written in English language. Additional studies were searched through the references of individual articles.

Data independently extracted from each article included age, male-to-female ratio, publication year, study design (prospective vs. retrospective), study country, number of participants, area under the receiver operating characteristic curve (AUROC) with 95% confidence intervals (CIs), sensitivity, specificity, and cut-off values used for the NLR and PLR. True positive (TP), false positive (FP), false negative (FN), and true negative (TN) values were either extracted or calculated according to the following formulas: Sensitivity = TP/(TP + FN); Specificity = TN/(FP + TN) [17].

Each article’s bias risk was assessed using the Joanna Briggs Institute Critical Appraisal Checklist for case–control studies [18]. We followed the PRISMA 2020 statement [19] (Supplementary Table 1) and registered the protocol in an international repository (PROSPERO registration number: CRD42024533546).

### Statistical analysis

The pooled sensitivity and specificity were calculated, and the corresponding forest plot was generated to assess the diagnostic accuracy of the NLR and PLR for the presence of RA and active disease [20]. Summary receiving characteristics (SROC) curves with 95% confidence region and prediction region were generated using the midas command [21]. The relationship between prior probability, likelihood ratio, and posterior test probability was assessed using Fagan’s nomogram plot [22]. Stata 14 was used for statistical analyses (StataCorp LLC, College Station, TX, USA).

## Results

### Study selection

**Fig. 1:**
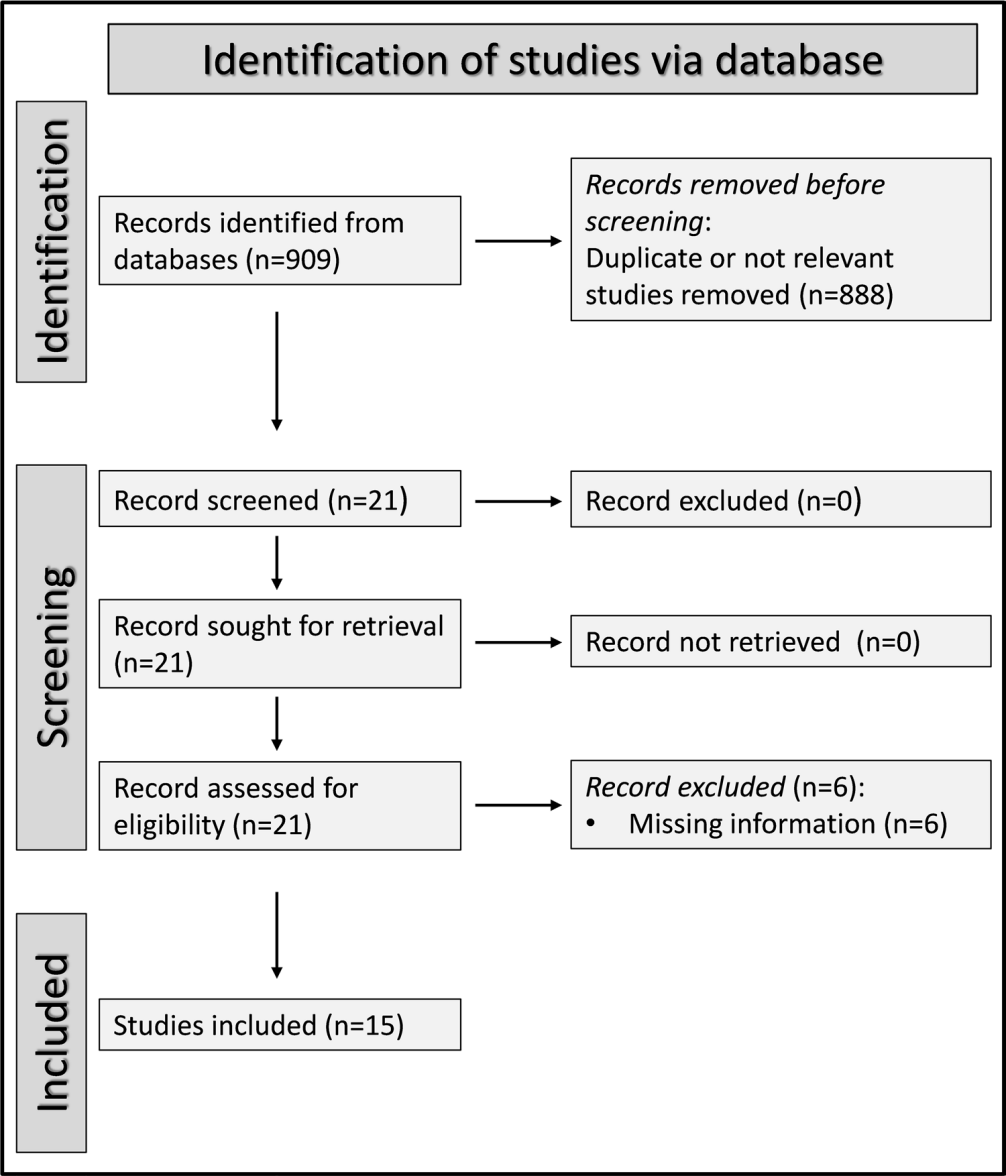
Flow chart of study selection

Our search strategy identified a total of 909 articles, of which 888 were excluded because they presented either duplicate data or irrelevant information. A comprehensive review of the remaining 21 articles led to the further exclusion of six studies due to missing data, leaving 15 studies for further analysis (Fig. 1 and Tables 1, 2) [23–37]. The risk of bias was assessed as low in all studies, barring one which exhibited moderate risk (Supplementary Table 2) [37].

**Table 1:** Characteristics of the studies investigating the diagnostic accuracy of the neutrophil-to-lymphocyte ratio and the platelet-to-lymphocyte ratio for the presence of rheumatoid arthritis

Study	Study design	N	Age (Years)	M/F	AUC (95% CI) NLR PLR	Cut-off NLR PLR	Sensitivity (%) NLR PLR	Specificity (%) NLR PLR
Peng YF et al., 2015, China [24]	P	219	54	71/148	NR 0.847 (0.794–0.901)	NR 115.66	NR 0.825	NR 0.748
Chen Q et al., 2019, [25]	P	488	61	101/387	NR 0.676 (NR)	NR 171.92	NR 0.6128	NR 0.8168
Erre GL et al., 2020, Italy [26]	P	346	55	123/223	0.67 (0.61–0.73) 0.65 (0.59–0.71)	1.88 141.4	0.618 0.462	0.693 0.857
Jin Z et al., 2021, China [29]	R	1254	64	239/1015	0.831 (NR) NR	2.13 NR	0.767 NR	0.759 NR
Lijuan W et al., 2021, China [31]	R	518	54	146/372	NR 0.869 (0.837–0.897)	NR NR	NR 0.9176	NR 0.7395
Song BW et al., 2022, South Korea [32]	R	341	58	NR	0.622 (NR) 0.58 (NR)	2.07 143.26	0.623 0.557	0.614 0.607
Xu Y et al., 2022, China [34]	R	1865	60	479/1386	0.847 (0.827–0.866) NR	2.28 NR	0.752 NR	0.817 NR
Obaid J et al., 2023, Yemen [37]	P	82	NR	NR	0.699 (0.571–0.819) NR	1.35 NR	0.574 NR	0.8 NR

Legend: NR, not reported; P, prospective; R, retrospective; M/F, male to female ratio; AUC, area under the curve; NLR, neutrophil-to-lymphocyte ratio; PLR, platelet-to-lymphocyte ratio

**Table 2:** Characteristics of the studies investigating the diagnostic accuracy of the neutrophil-to-lymphocyte ratio and the platelet-to-lymphocyte ratio for the presence of active disease

Study	Study design	N	Age (Years)	M/F	AUC (95% CI) NLR PLR	Cut-off NLR PLR	Sensitivity (%) NLR PLR	Specificity (%) NLR PLR
Chandrashekara S et al., 2015 India [23]	P	88	50	12/76	0.584 (0.474–0.688) NR	2.9 NR	0.3043 NR	0.8 NR
Remalante PPM et al., 2020, Philippines [27]	R	134	56	2/132	0.629 (NR) NR	2.32 NR	0.546 NR	0.769 NR
Dechanuwong P et al., 2021, Thailand [28]	R	325	55	38/287	0.592 (0.527–0.657) NR	2.6 NR	0.752 NR	0.432 NR
Lijuan W et al., 2021, China [30]	R	547	56	102/445	0.6 (NR) 0.597 (NR)	4.5 167.5	0.318 0.573	0.778 0.639
Taha SI et al., 2022, Egypt [33]	P	100	67	19/81	0.69 (0.521–0.858) NR	3.3 NR	0.782 NR	0.538 NR
El-Husseiny PN et al., 2023, Egypt, [35]	P	47	44	4/43	0.78 (0.68–0.82) 0.841 (0.6–0.84)	1.52 121.9	0.842 0.789	0.442 0.442
Elsayed SA et al., Egypt, 2023 [36]	P	120	43	12/108	0.913 (NR) 0.841 (NR)	1.3 112.39	0.869 0.859	0.81 0.67

Legend: NR, not reported; P, prospective; R, retrospective; M/F, male to female ratio; AUC, area under the curve; NLR, neutrophil-to-lymphocyte ratio; PLR, platelet-to-lymphocyte ratio

### Neutrophil-to-lymphocyte ratio

#### Presence of rheumatoid arthritis

Five studies assessing a total of 3888 subjects (2830 RA patients and 1058 healthy controls, 78% females, mean age 60 years) reported the sensitivity and specificity of the NLR for the presence of RA [26, 29, 32, 34, 37] (Table 1). Two studies were performed in China [29, 34], one in Italy [26], one in Korea [32], and one in Yemen [37]. Three studies were retrospective [29, 32, 34], and the remaining two prospective [26, 37].

**Fig. 2:**
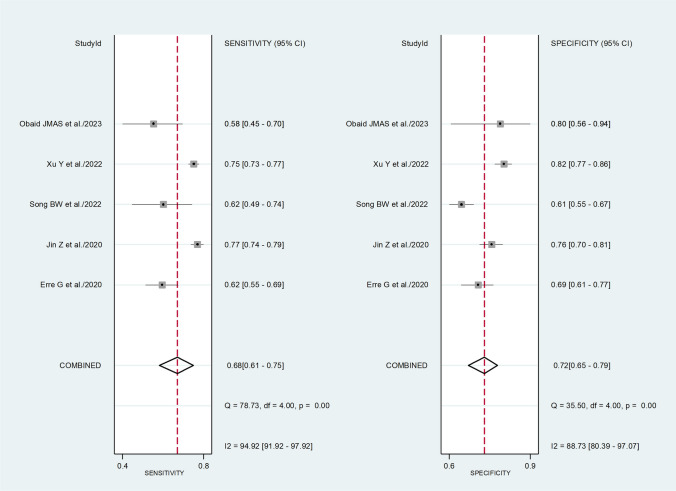
Forest plot of the pooled estimates of sensitivity and specificity of the neutrophil-to-lymphocyte ratio for the presence of rheumatoid arthritis

**Fig. 3:**
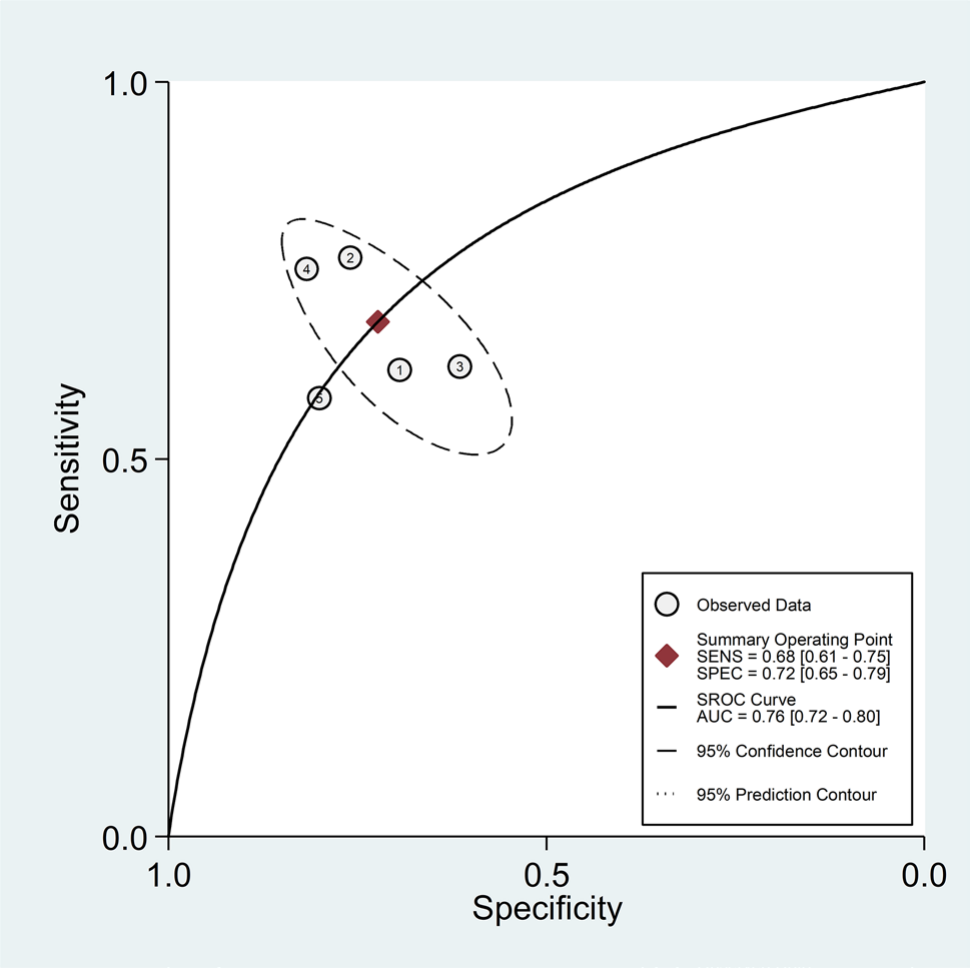
Summary receiver operating characteristics curve with 95% confidence region and prediction region of the neutrophil-to-lymphocyte ratio for the presence of rheumatoid arthritis

The pooled sensitivity and specificity for the presence of RA were 0.68 (95% CI 0.61 to 0.75) and 0.72 (95% CI 0.65 to 0.79), respectively (Fig. 2). The SROC curve with 95% confidence region and prediction region showed an AUC value of 0.76 (95% CI 0.72 to 0.80), with the summary operating point at sensitivity of 0.68 and specificity of 0.72 (Fig. 3). Assessment of publication bias and meta-regression analysis could not be performed because of the small number of studies.

**Fig. 4:**
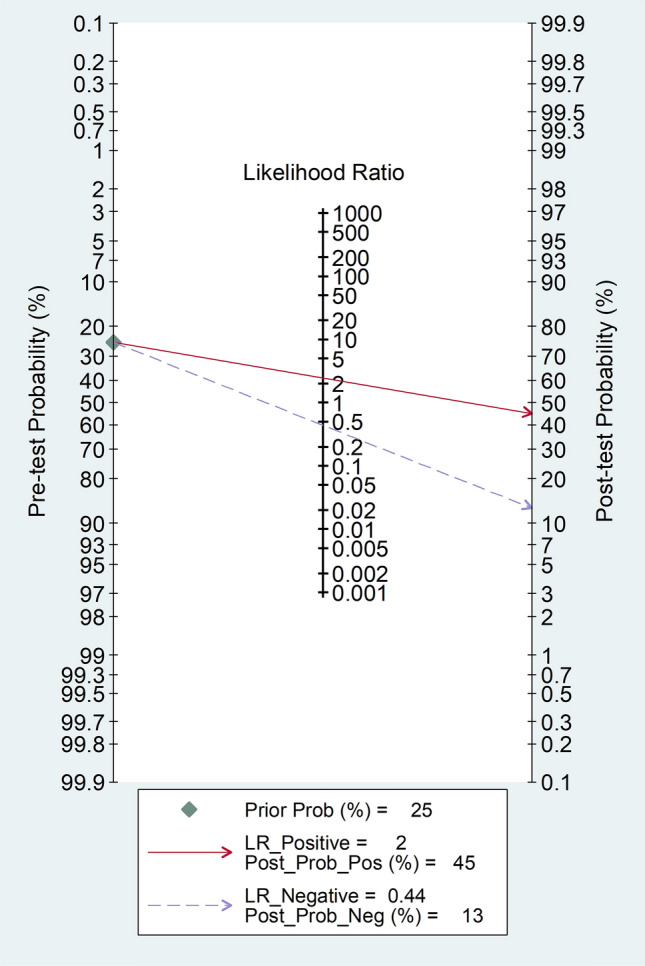
Fagan’s nomogram of the neutrophil-to-lymphocyte ratio for the presence of rheumatoid arthritis

Fagan’s nomogram showed that, assuming a pre-test probability of RA of 25%, the post-test probability was 45% in subjects with relatively high NLR values and 13% in those with relatively low NLR values (Fig. 4).

### Active disease

Seven studies assessing a total of 1361 RA patients (933 with active disease, 428 with non-active disease, 86% females, mean age 55 years) reported the sensitivity and specificity of the NLR for active disease [23, 27, 28, 30, 33, 35, 36] (Table 2). Three studies were performed in Egypt [33, 35, 36], one in China [30], one in India [23], one in the Philippines [27], and one in Thailand [28]. Three studies were retrospective [27, 28, 30], whilst four were prospective [23, 33, 35, 36]. In all studies, disease activity was assessed using the DAS28 and a threshold of 2.6 was used to define the presence of active disease.

**Fig. 5:**
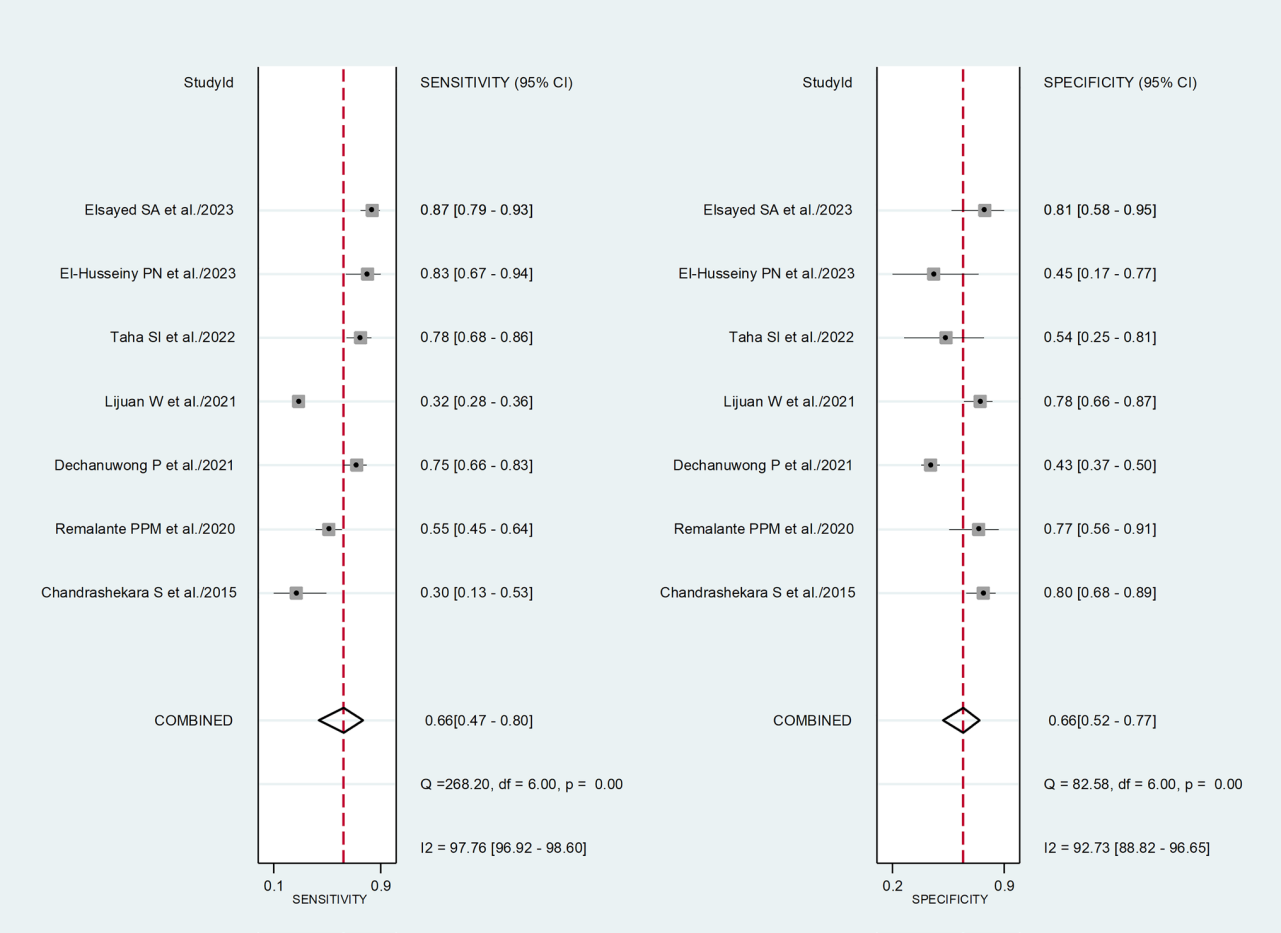
Forest plot of the pooled estimates of sensitivity and specificity of the neutrophil-to-lymphocyte ratio for the presence of active disease

**Fig. 6:**
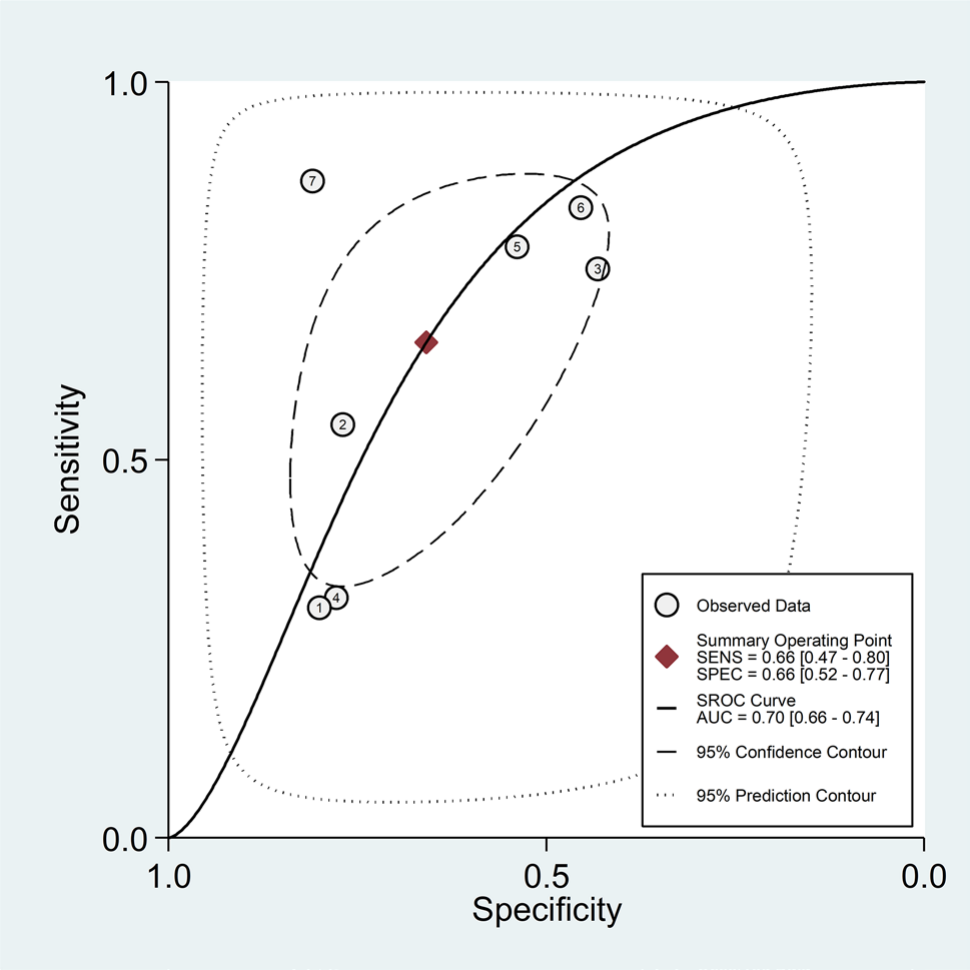
Summary receiver operating characteristics curve with 95% confidence region and prediction region of the neutrophil-to-lymphocyte ratio for the presence of active disease

The pooled sensitivity and specificity of the NLR for active disease were 0.66 (95% CI 0.47 to 0.80) and 0.66 (95% CI 0.52 to 0.77), respectively (Fig. 5). The SROC curve with 95% confidence region and prediction region showed an AUC value of 0.70 (95% CI 0.66 to 0.74) with the summary operating point at sensitivity of 0.66 and specificity of 0.66 (Fig. 6). Assessment of publication bias and meta-regression analysis could not be performed because of the small number of studies.

**Fig. 7:**
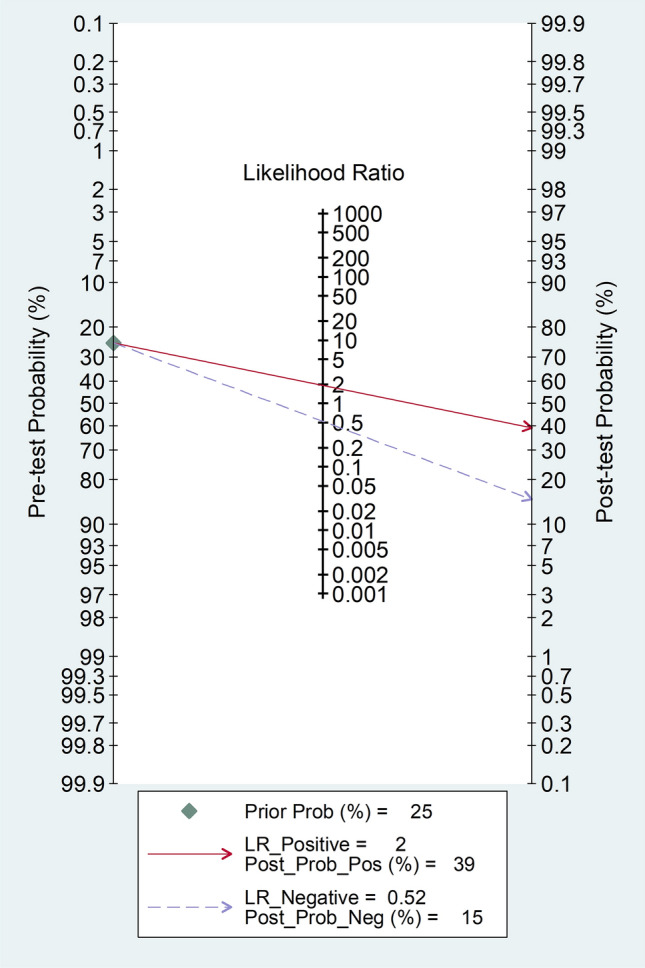
Fagan’s nomogram of the neutrophil-to-lymphocyte ratio for the presence of active disease

Fagan’s nomogram showed that, assuming a pre-test probability of active disease of 25%, the post-test probability was 39% in subjects with relatively high NLR values and 15% in those with relatively low NLR values (Fig. 7).

### Platelet-to-lymphocyte ratio

#### Presence of rheumatoid arthritis

Five studies investigating a total of 1912 subjects (777 RA patients and 1135 healthy controls, 77% females, mean age 57 years) reported the sensitivity and specificity of the PLR for the presence of RA [24–26, 31, 32] (Table 1). Three studies were conducted in China [24, 25, 31], one in Italy [26], and one in Korea [32]. Two studies were retrospective [31, 32], and the remaining three prospective [24–26].

**Fig. 8:**
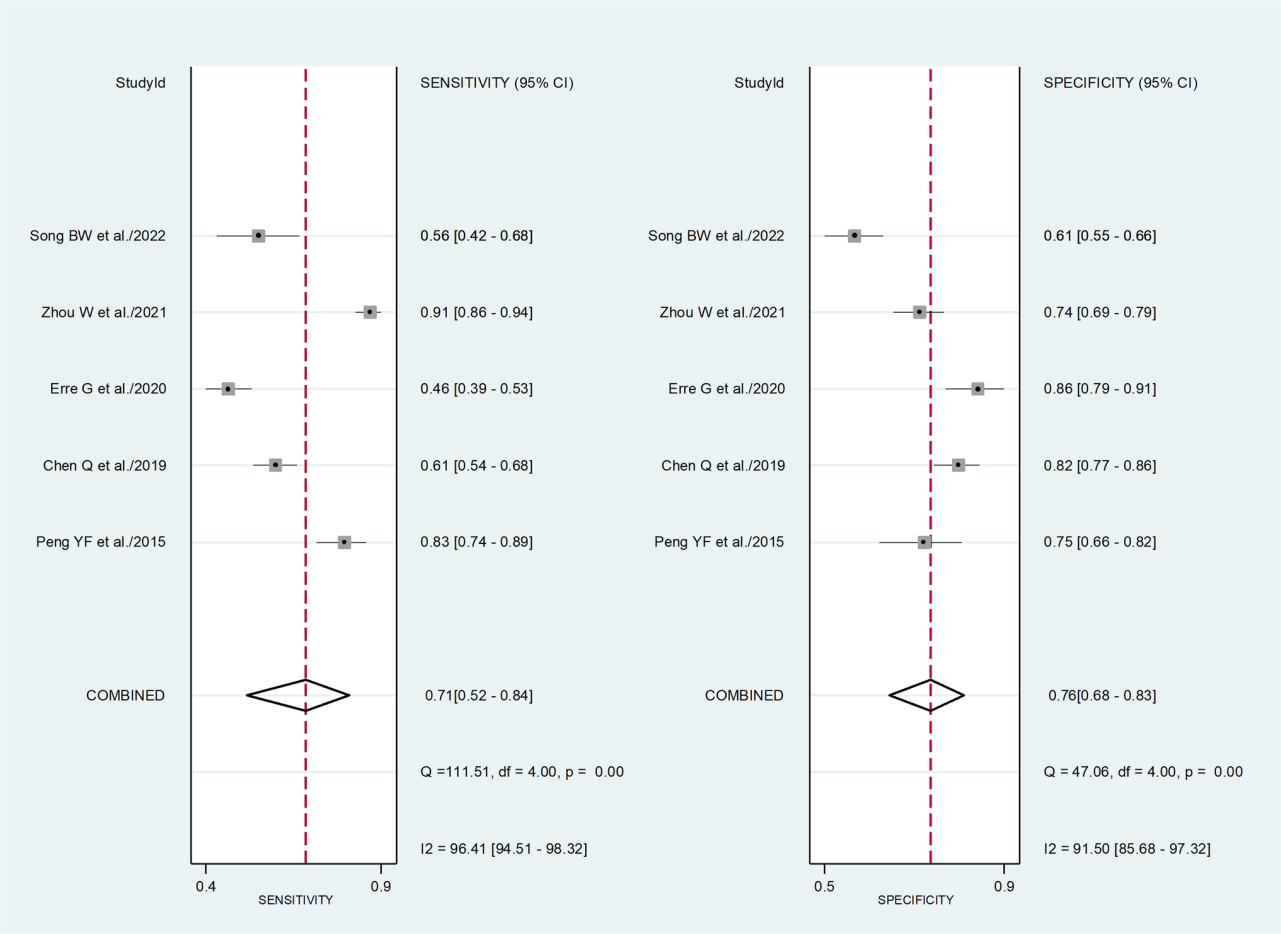
Forest plot of the pooled estimates of sensitivity and specificity of the platelet-to-lymphocyte ratio for the presence of rheumatoid arthritis

**Fig. 9:**
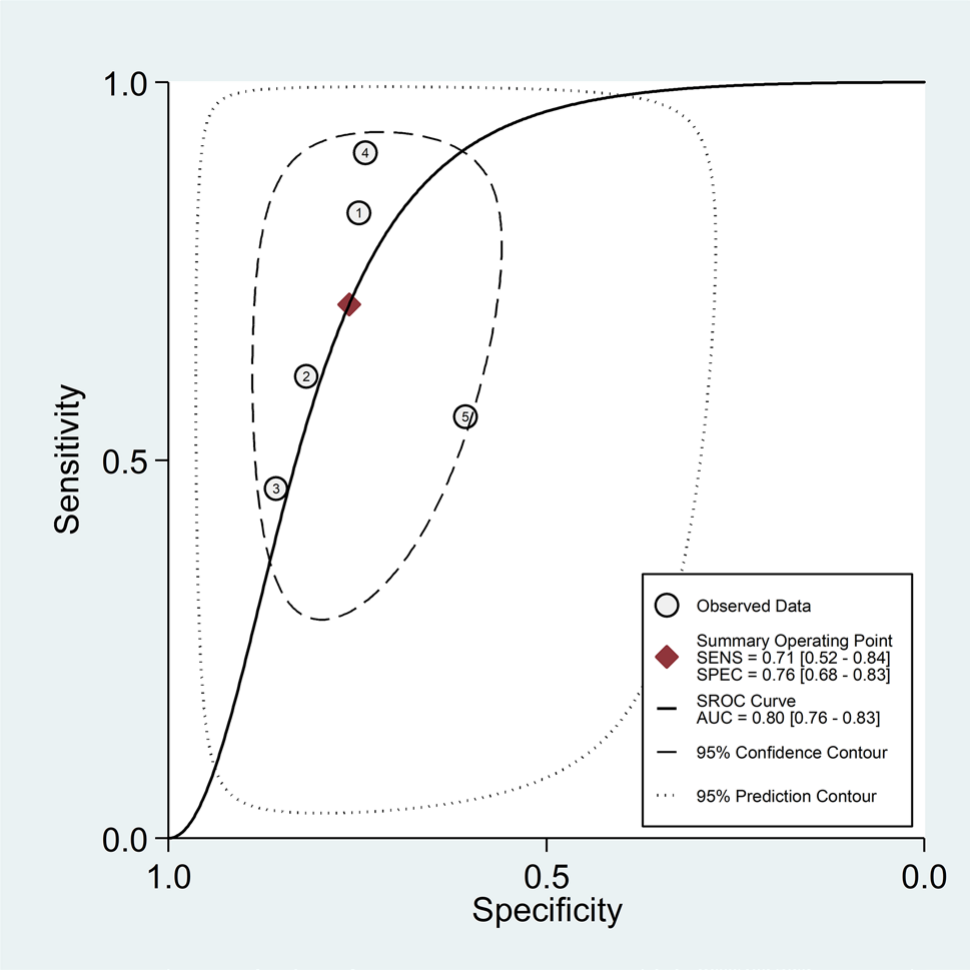
Summary receiver operating characteristics curve with 95% confidence region and prediction region of the platelet-to-lymphocyte ratio for the presence of rheumatoid arthritis

The pooled sensitivity and specificity of the PLR for the presence of RA were 0.71 (95% CI 0.52 to 0.84) and 0.76 (95% CI 0.68 to 0.83), respectively (Fig. 8). The SROC curve with 95% confidence region and prediction region showed an AUC value of 0.80 (95% CI 0.76 to 0.83), with the summary operating point at sensitivity of 0.71 and specificity of 0.76 (Fig. 9). Assessment of publication bias and meta-regression analysis could not be performed because of the small number of studies.

**Fig. 10:**
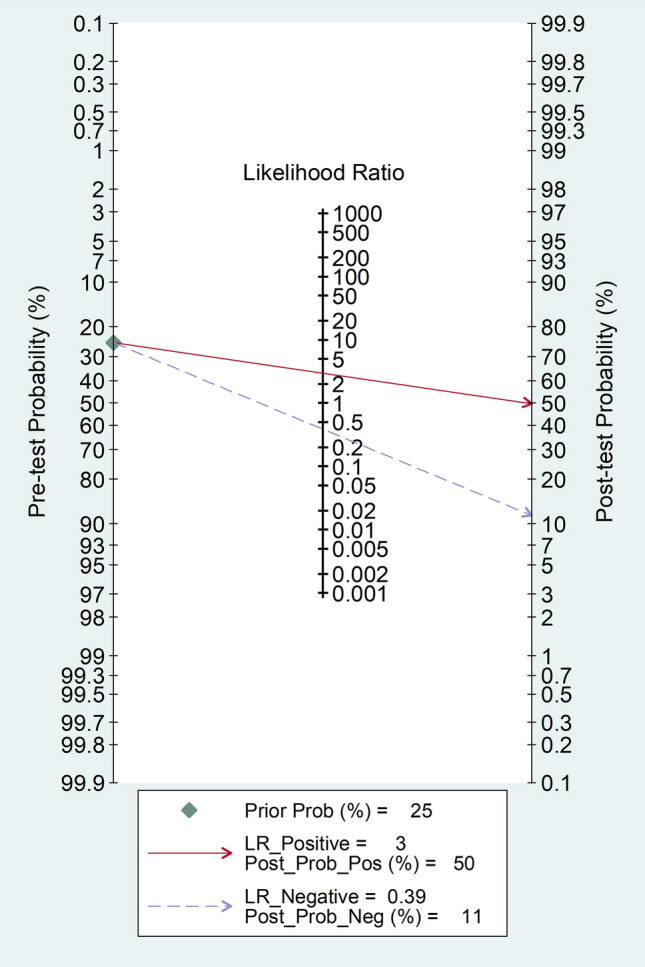
Fagan’s nomogram of the neutrophil-to-lymphocyte ratio for the presence of rheumatoid arthritis

Fagan’s nomogram showed that, assuming a pre-test probability of RA of 25%, the post-test probability was 50% in subjects with relatively high PLR values and 11% in those with relatively low PLR values (Fig. 10).

### Active disease

Three studies in a total of 714 RA patients (610 with active disease, 104 with non-active disease, 83% females, mean age 53 years) reported the sensitivity and specificity of the PLR for active disease [30, 35, 36] (Table 2). Two studies were conducted in Egypt and were prospective [35, 36], whereas the third one was conducted in China and was retrospective [30]. In all studies, disease activity was assessed using the DAS28 and a threshold of 2.6 was used to define the presence of active disease.

Forest plots for pooled sensitivity and specificity and the SROC curve could not be generated, given the limited number of studies. Lijuan W et al. reported an AUC value of 0.597 with 0.576 sensitivity and 0.639 specificity [30]. El-Husseiny PN et al. reported an AUC of 0.72 with 0.789 sensitivity and 0.442 specificity [35]. Elsayed SA et al. reported an AUC of 0.841 with 0.859 sensitivity and 0.67 specificity [36].

## Discussion

Taken together, the results of our study suggest that haematological indices derived from routine full blood cell counts can be helpful in diagnosing RA and the presence of active disease. Based on the observed AUC [8], the NLR exhibited a moderate diagnostic accuracy for the presence of RA and active disease, whereas the PLR exhibited a good diagnostic accuracy for the presence of RA. There were insufficient studies to assess the accuracy of the PLR for the presence of active disease. These observations support the potential clinical use of the NLR and the PLR, combined with clinical assessment and other serological biomarkers, in diagnosing and managing patients with RA.

A recent systematic review and meta-analysis of 19 studies investigating the diagnostic accuracy of the RF for the presence of RA (4786 RA patients and 6994 controls) reported an AUC for SROC curve of 0.85 (standard error = 0.03). However, pooled analysis of the prognostic accuracy was not performed, given the significant between-study differences in the assessed endpoints (disease activity, radiographic progression, and treatment response) [38]. In another systematic review and meta-analysis of 24 studies investigating the diagnostic accuracy for the presence of RA in 7344 participants, the AUC for SROC curve for anti-CCP or RF was 0.87 (standard error = 0.01) [39]. However, half of the selected studies included patients with other rheumatic diseases as control group, no study used healthy controls as control group, and no assessment of the diagnostic accuracy for active disease was conducted. Further studies are therefore necessary to adequately compare the diagnostic accuracy of the NLR, PLR, RF, and anti-CCP, singly or in combination, in RA patients and control groups with similar characteristics. As previously discussed, the AUC values of the NLR and PLR reported in our systematic review and meta-analysis compare favourably with other non-specific biomarkers of inflammation, i.e., C-reactive protein and erythrocyte sedimentation rate [7, 8].

The potential clinical use of the NLR and PLR is further supported by the information provided by Fagan’s nomogram, which showed a tangible separation in the probability of having RA or active disease given a relatively high NLR and/or PLR. However, these results should prompt the conduct of appropriately designed prospective studies to investigate the added benefits of measuring the NLR and PLR in patients with RA from a clinical and health economics point of view. Such studies should assess the influence of several factors potentially influencing the diagnostic accuracy of these haematological indices. These factors include the use of standard cut-off values and specific clinical and demographic patient characteristics likely to account for the variability in early clinical presentation and disease progression [40–42]. These issues notwithstanding, the cost-effectiveness of measuring the NLR and PLR appears particularly appealing in the evaluation and monitoring of RA patients, given the routine evaluation of full blood cell counts and individual cell types in this group [43].

An additional issue worth investigating is the relative weight and place of measuring the NLR and/or PLR within current algorithms and scoring systems for the diagnosis of RA. For example, the 2010 American College of Rheumatology/European League Against Rheumatology (ACR/EULAR) criteria are primarily based on the number and site of involved joints, elevations of RF and/or anti-CCP, elevations in acute phase reactants (C-reactive protein and erythrocyte sedimentation rate), and symptom duration [44]. Further research is warranted to determine whether the NLR and/or PLR should be measured before, during, or after the assessment of RF, CCP, and acute phase reactants to enhance diagnostic accuracy. Similarly, studies should investigate the ideal place for NLR and/or PLR assessment within current recommendations for disease monitoring and evaluation of treatment response [45].

Neutrophils, platelets, and lymphocytes are involved in the pathophysiology and clinical manifestations of RA. Neutrophils play a critical role in driving inflammatory processes not only in the early stages of RA but also in the progression of the disease. These cells are the most abundant cell type in the synovial fluid of patients with RA, and the elevated expression of chemokines further augments the inflammatory response locally and systemically [46]. Furthermore, neutrophils are responsible for the overproduction of reactive oxygen species, with the consequent alteration of the redox balance, and the formation of neutrophil extracellular traps [47–49]. Similarly, there is a complex interplay between platelet alterations, inflammation, and disease activity in RA as platelets release several inflammatory mediators such as cytokines, chemokines, and growth factors [50–53]. Furthermore, the presence of platelet microparticles in the circulation of RA patients exerts a significant pro-coagulant effect and, at the same time, expresses autoantigens that perpetuate the generation of pro-inflammatory immune complexes involved in synovial inflammation [54, 55]. While a reduction in the lymphocyte count is commonly observed in RA as well as other autoimmune conditions [56], functional alterations of this cell type have been increasingly reported in RA, including dysregulated proliferation and differentiation and excessive inflammatory responses [57, 58]. Further research is required to investigate whether alterations in neutrophil, platelet, and lymphocyte counts, reflected in the modifications of the NLR and PLR, are associated with functional alterations of these cell types and with the clinical manifestations of RA.

One strength of our systematic review and meta-analysis was the comprehensive assessment of the sensitivity and specificity of the NLR and PLR for the presence of RA and active disease, assessed using the AUC and the Fagan’s nomogram, which provides essential information regarding the diagnostic accuracy of these haematological indices. One significant limitation was that all identified studies, except one [26], were conducted in Africa and Asia, which prevents the generalisability of our findings and requires further studies in other geographical locations given the presence of ethnic-related differences in the NLR and PLR in other patient groups [59–62]. A further limitation is the lack of information in our analyses regarding the possible direct influence of common features in RA patients, particularly infections and co-morbidities [63–65], and pharmacological treatments [66, 67], on these haematological indices and, consequently, their diagnostic accuracy.

In conclusion, the results of our systematic review and meta-analysis suggest that the NLR and the PLR have promising diagnostic accuracy for the presence of RA and active disease. However, their routine use in clinical practice requires confirmation and validation in prospective studies investigating the added benefits of these haematological indices within existing algorithms, and the potential influence of concurrent infections, comorbidities, and specific treatments, in diagnosing and managing patients with RA.

## Supplementary Information

Below is the link to the electronic supplementary material.

Supplementary file1 (DOCX 34 KB)

Supplementary file2 (DOCX 17 KB)

## Data Availability

The data that support the findings of this systematic review and meta-analysis are available from AZ upon reasonable request.
