# Supplementary material for: Diagnostic accuracy of the neutrophil-to-lymphocyte ratio and the platelet-to-lymphocyte ratio in rheumatoid arthritis: a systematic review and meta-analysis
Source: Clin Exp Med. 2024 Sep 4;24(1):207. doi: 10.1007/s10238-024-01478-x (PMC11374877; doi:10.1007/s10238-024-01478-x)
Supplement: Supplementary file 2 — Supplementary file2 (DOCX 17 KB) [file 10238_2024_1478_MOESM2_ESM.docx]

**Supplementary Table 2.** Assessment of the risk of bias using the Joanna Briggs Institute critical appraisal checklist.

| **Study** | **Were the inclusion criteria clearly defined?** | **Were the subjects and the setting described in detail?** | **Was the exposure measured in a reliable way?** | **Were standard criteria used to assess the condition?** | **Were confounding factors identified?** | **Were strategies to deal with confounding factors stated?** | **Were the outcomes measured in a reliable way?** | **Was appropriate statistical analysis used?** | **Risk of bias** |
| --- | --- | --- | --- | --- | --- | --- | --- | --- | --- |
| Peng et al., 2015 | Yes | Yes | Yes | Yes | No | No | Yes | Yes | Low |
| Chen et al., 2019 | Yes | Yes | Yes | Yes | Yes | Yes | Yes | Yes | Low |
| Erre et al., 2020 | No | Yes | Yes | Yes | Yes | Yes | Yes | Yes | Low |
| Jin et al., 2021 | Yes | Yes | Yes | Yes | Yes | Yes | Yes | Yes | Low |
| Zhou et al., 2021 | Yes | Yes | Yes | Yes | No | No | Yes | Yes | Low |
| Song et al., 2022 | Yes | Yes | Yes | Yes | No | No | Yes | Yes | Low |
| Xu et al., 2022 | Yes | Yes | Yes | Yes | Yes | Yes | Yes | Yes | Low |
| Obaid et al., 2023 | No | Yes | Yes | Yes | No | No | Yes | Yes | Moderate |
| Chandrashekara et al., 2015 | Yes | Yes | Yes | Yes | No | No | Yes | Yes | Low |
| Remalante et al., 2020 | Yes | Yes | Yes | Yes | No | No | Yes | Yes | Low |
| Dechanuwong et al, 2021 | Yes | Yes | Yes | Yes | Yes | Yes | Yes | Yes | Low |
| Lijuan et al., 2021 | Yes | Yes | Yes | Yes | No | No | Yes | Yes | Low |
| Taha et al., 2022 | Yes | Yes | Yes | Yes | No | No | Yes | Yes | Low |
| El-Husseiny et al., 2023 | Yes | Yes | Yes | Yes | No | No | Yes | Yes | Low |
| Elsayed et al., 2023 | Yes | Yes | Yes | Yes | No | No | Yes | Yes | Low |
